# The Effects of City Lockdown Policy on Frozen Embryo Transfer Outcome during COVID-19 Epidemic in Hubei Province, China

**DOI:** 10.1155/2022/8662279

**Published:** 2022-04-13

**Authors:** Huiqi Liao, Zhifeng Sun

**Affiliations:** ^1^Reproductive Medicine Center, Renmin Hospital, Hubei University of Medicine, Shiyan 442000, China; ^2^Hubei Clinical Research Center for Reproductive Medicine, Shiyan 442000, China; ^3^Biomedical Engineering College, Hubei University of Medicine, Shiyan 442000, China; ^4^Biomedical Research Institute, Hubei University of Medicine, Shiyan 442000, China; ^5^Hubei Key Laboratory of Embryonic Stem Cell Research, Shiyan 442000, China

## Abstract

**Objective:**

To evaluate whether there is a difference in clinical pregnancy rate and live birth rate between the corresponding period in 2019 and COVID-19 city lockdown period in 2020 in frozen embryo transfer (FET).

**Methods:**

In one single *in vitro* fertilization (IVF) center (Shiyan, Hubei province, China), a retrospective cohort analysis was conducted, with a sample size of 59 patients in the lockdown period (2020.1.23-2020.2.23, 2020 group) and 34 patients in the corresponding 2019 period (2019.1.23-2019.2.23, 2019 group). Implantation, biochemical and clinical pregnancy, miscarriage, and live birth rates were all measured.

**Results:**

Age, basal serum follicle-stimulating hormone (FSH), basal serum luteinizing hormone (LH), basal serum E2, and serum total T were all comparable between the two groups. On the day of progesterone administration, endometrial thickness was similar (8.5 ± 1.3 vs. 8.2 ± 1.4, *P* = 0.356). The number of transferred blastocysts was not significantly different. The two groups had similar clinical pregnancy rate (61.8% vs. 61.0%, *P* > 0.05) and live birth rate (47.1% vs. 49.2%, *P* > 0.05), which did not significantly differ. Nonetheless, there was a significant difference in the cancelled cycle rate between the two groups (0% vs. 28.0%, *P* = 0.043).

**Conclusions:**

Lockdown period FET versus corresponding period FET outcome did not show any significant difference in terms of pregnancy rate and live birth rate between two groups of patients. Although there was no significant difference, in the 2020 group, the live birth rate was higher compared with that in the 2019 group. There was a significant difference in the rate of cancelled cycles due to the seal off control. In summary, artificial endometrial preparation is an appropriate protocol for special periods.

## 1. Introduction

When the whole nation was preparing for the Spring Festival in 2020, an outbreak of severe acute pneumonia emerged in Wuhan, Hubei Province, China [1–3]. On February 12, 2020, the World Health Organization (WHO) officially named the disease caused by the novel coronavirus as coronavirus disease 2019 (COVID-19). The virus was spread throughout the world via people's travels [4]. Anxiety, panic attacks, school closure, and social isolation are some consequences of the COVID-19 pandemic. Control measures were implemented in China and other nations to limit the spread of the disease. On 23rd and 24th of January, Wuhan and its adjacent cities were placed under the metropolitan-wide quarantine. Furthermore, beginning at 10 a.m. on January 23rd, public transportation was suspended in Shiyan, and everyone must be quarantined at his/her home.

During this period, not only related departments were affected but also reproductive centers were influenced, all COH cycles had to cancel, most of the frozen embryo transfer (FET) cycles were not completed, and patients were instructed by doctors through telephone or WeChat platform to learn how they can use medicines. In the present study, it was attempted to indicate whether there is a difference in pregnancy and live birth rates between the lockdown period (2020.1.23-2020.2.23) and 34 patients in the corresponding 2019 period (2019.1.23-2019.2.23) in FET cycles.

## 2. Materials and Methods

A total of 82 FET cycles were recorded in the *in vitro* fertilization (IVF) center Shiyan, Hubei province, China, between January 23, 2020, and February 23, 2020, i.e., the city lockdown period. Besides, 34 FET cycles of the corresponding period of 2019 were analyzed. The inclusion criteria were as follows: age < 40 years, body mass index (BMI) < 30 kg/m^2^, a regular menstrual cycle, a history of assisted reproductive technology (ART) (the number of trials ≤ 2), and a history of fertilized oocytes (the number of trials > 3). The exclusion criteria were as follows: uterine deformities, hyperprolactinemia, thyroid abnormalities, ovulation abnormalities, a history of recurrent miscarriage, tuberculosis, and severe endometriosis. The Institutional Review Board of Renmin Hospital (Shiyan, China) approved the study protocol.

### 2.1. Stimulation of the Ovaries, Oocyte Collection, and Embryonic Culture

The gonadotropin-releasing hormone (GnRH) agonist (3.75 mg, Diphereline, Beaufour-Ipsen) protocol was used, with ovarian stimulation using recombinant follicle-stimulating hormone (150–225 IU rFSH, Gonal-F; Merck Serono, Darmstadt, Germany). When three or more follicles had grown to a diameter of 18 mm, recombinant human chorionic gonadotropin (rHCG; 250 *μ*g; Merck Serono) was injected or a combination of gonadotropin-releasing hormone agonist (GnRHa), and rHCG (2000 U) was used to trigger final oocyte maturation. Transvaginal ultrasonography- (TVS-) guided follicular aspiration was used to collect oocytes at 36 h after triggering. The incubation was conducted under the conditions of 6% CO_2_, 5% O_2_, and 37.0° C. IVF/intracytoplasmic sperm injection (ICSI) was used to perform oocyte insemination, accompanying by daily embryonic assessment.

### 2.2. Cryopreservation of Blastocysts

Ultrarapid technology (Cryotop, Kitazato BioPharma Co. Ltd., Fuji City, Japan) was used to vitrify and thaw blastocysts, according to the manufacturer's instructions. On the same day, blastocysts were warmed and transferred; blastocysts were incubated for 2 h after warming before being morphologically assessed. Afterward, any blastocysts that were not selected for transfer were revitrified.

### 2.3. Endometrial Preparation

In the artificial cycle, estradiol valerate (Progynova; Bayer Inc., Leverkusen, Germany) was orally given in a step-up regimen of 4 mg/day from day 1 to day 10, followed by 6-8 mg/day from day 11 to day 16. On the 10^th^ and 16^th^ days of estrogen administration, an ultrasound scan was carried out to determine endometrial thickness, and serum progesterone test was then conducted. Estrogen was persisted at 8 mg/day if the endometrium was denser than 7 mm and serum progesterone level was significantly lower than 2 ng/mL, and daily progesterone injection (90 mg/day; Crinone VR 8%; Merck Serono) was started on day 16 (or on the day after confirmation of adequate endometrial thickness). On the 6^th^ day of progesterone injection, a vitrified-warmed FET was conducted.

GnRH-a (3.75 mg, Diphereline; Ipsen, Paris, France) was administered at a subcutaneous daily dose of 3.75 mg on the 2^nd^ day of the menstruation in the artificial cycle with downregulation. An ultrasound scan and E2 measurement were conducted 35 days later to confirm pituitary sensitization, and endometrial preparation was started using the artificial cycle protocol if endometrial thickness would be <5 mm and blood estradiol level would be slightly <50 pg/ml.

In the lockdown time during the COVID-19 epidemic in Hubei province, patients could not finish the endometrial preparation as planned, they were directed to use medicines according to physicians' prescription provided through telephone or WeChat platform, and they completed endometrial preparation under physicians' guidance based on the abovementioned protocol. Ultrasound was conducted on the 16^th^ day of estrogen administration to quantify endometrial thickness, and serum progesterone level was quantified.

### 2.4. FET

In patients with a full bladder, an aural window was provided for imaging of the uterus for cavity measurement and supraclavicular ET. All ETs were carried out using a Cook catheter (Soft Pass, J-SPPE; Cook Ob/Gyn, Spencer, IN, USA), and the abdominal ultrasonography was performed with a 5 MHz probe by an ultrasonographer (Sonoline, Adara; Siemens, Munich, Germany). The embryos were inserted into the tip of the catheter, which was then placed at a depth of 1.0–2.0 cm under the peak of the endometrial cavity as determined by transabdominal ultrasonography. Patients lied in bed for 10 min after the embryo transfer. During the previous 12 months, two operators in this trial achieved identical results (approximately 2,000 transfers).

### 2.5. Luteal Supplementation

Vaginal progesterone (90 mg/day, Crinone VR 8%; Merck Serono) was used to supplement the luteal phase, which began before the embryo transfer and lasted for 14 days. Patients were instructed to continue luteal support until the 8–10^th^ gestational weeks if their serum HCG level was >30 IU/L at 14 days after ET. The same dose of estradiol was given to both groups until the 8–10^th^ weeks of pregnancy, and the dose was gradually reduced.

### 2.6. Outcome Measures

The outcomes included implantation, biochemical and clinical pregnancy, miscarriage, and live birth rates. On the day of embryo transfer for 14 days, if the blood b-HCG level was >30 IU/L, patients were considered as biochemical pregnancy. The number of visible gestational sacs was divided by the number of embryos transferred for each patient to calculate the implantation rate. The presence of a gestational sac with fetal heart rate on ultrasound was defined as clinical pregnancy. The spontaneous loss of clinical pregnancy was defined as miscarriage. Cancellation cycle rate was calculated as the number of cancellation cycles/total number of cycles.

### 2.7. Statistical Analysis

In the present study, SPSS 20.0 software was used to perform statistical analysis (IBM Corp., Armonk, NY, USA). The normally distributed continuous variables were expressed as mean ± standard deviation; nonnormally distributed data were presented as median (interquartile range (IQR)). The *t*-test was used to compare normally distributed data between groups, while the Mann–Whitney *U* test was employed to compare nonnormally distributed data. The Chi-square (*χ*^2^) test was utilized to compare qualitative data between groups. *P* < 0.05 was considered statistically significant.

## 3. Results

Among 105 female patients, only 93 female patients met the study's requirements and were involved in the present study. In the 2019 group, all 34 patients underwent FET. In the 2020 group, among 82 patients, 23 patients did not undergo FET because of the seal off control. The final analysis of pregnancy and live birth rates contained 34 and 59 cycles in 2019 and 2020 groups, respectively.

Patients in both groups had similar demographic characteristics (Table 1). Age (30.88 ± 4.04 vs. 32.83 ± 5.05, *P* = 0.059) and BMI (22.75 ± 3.74 vs. 23.50 ± 3.43, *P* = 0.336) were similar between groups. In terms of basal serum FSH, basal serum luteinizing hormone (LH), basal serum E2, and serum total T, there were no significant differences between the two groups. On the day of progesterone administration, endometrial thickness was similar (8.5 ± 1.3 vs. 8.2 ± 1.4, *P* = 0.356). The number of embryos transferred did not significantly differ.

Table 2 compares the results of the FET cycles between the two groups. Biochemical pregnancy rate (70.6 vs. 72.9%, *P* = 0.647), clinical pregnancy rate (61.8 vs. 61.0%, *P* = 0.892), and live birth rate (47.1 vs. 49.2%, *P* = 0.681) were not significantly different between the two groups. However, there was a significant difference in the cancelled cycle rate between the two groups (embryos that did not survive after FET were excluded) (0 vs. 28.39%, *P* = 0.043).

## 4. Discussion

During the blockade of the city caused by the COVID-19 outbreak, the most important challenge for patients is not only the pandemic but also the inability to timely and accurately evaluate the endometrial preparation and to adjust the medication. Hence, they felt nervous and anxious and worried about pregnancy outcomes. Patients could not finish the endometrial preparation as the planned protocol, and they had to be directed by doctors to learn how to use the medicines through telephone or WeChat. Therefore, the main question in the current study was whether there would be any difference in pregnancy and live birth rates between 2019 year and COVID-19 seal off period in FET.

The proper synchronization between endometrial preparation and embryo developmental potential determines the outcome of a FET protocol [5]. In the present study, to exclude the influence of embryo quality on pregnancy outcomes, patients with blastocyst transfer were selected. The data showed that in 2020 and 2019 groups, clinical pregnancy rate was similar (61.8% vs. 61%), live birth rate was higher in 2020 group (49.2% vs. 47.1%), age was younger in the 2020 group (32.83 vs. 30.88), and average transfer embryo was 1.57 versus 1.47, indicating that blockade policy did not affect pregnancy outcomes. This is because all of the FET cycles were generated following artificial endometrial preparation with exogenous estrogen and/or pituitary downregulation with GnRH agonist.

The cycle is monitored for ovulation using blood tests or ultrasound to determine the thickness and maturation of the endometrium in a natural FET cycle (when no drugs are used before the embryo transfer) [6]. In women who have regular menses, FET must be coincided with ovulation in the natural cycle or after artificially prepping the endometrium with estrogen and progesterone. Estradiol and progesterone are supplied in a sequential regimen in an artificial cycle to imitate the endometrium's endocrine exposure during the regular cycle. Initially, estradiol is provided to cause endometrial proliferation, while hinders the development of dominant follicle. This can be continued until the endometrial thickness reaches 7 mm or more, when progesterone is administered to start the secretory transition [7]. Hill et al. (2010) found that using synthetic hormones for FET cycles produces better outcomes than using natural hormones [8].

Similar results were obtained in the present study, especially for the blockade period, in which patients must isolate at home, blood tests or ultrasound used to determine the thickness and maturity of the endometrium were not applicable to follow ovulation, and a doctor directed HT protocol via an online platform with or without GnRHa that prepared the endometrium easier and better. They recommended estradiol step-up strategy rather than high-dose estrogen, because former is more comparable to physiological patterns and is better for endometrial development and ultimately embryo implantation [8]. In this present research, patients who were directed by a doctor utilized a step-up regimen for endometrial preparation.

Endometrial receptivity may require an endometrial thickness of 5-8 mm [5]. A lower dose results in a higher rate of abortion. For the optimal growth of progesterone receptors and transition to implantation-ready endometrium, adequate endometrial proliferation is essential [9].

Pretreatment with GnRHa, according to some researchers, is beneficial in preventing spontaneous ovulation and cycle cancellation. Several researches evaluated the two artificial procedures, whether they used a GnRH agonist or not, and found that the reproductive outcomes were similar [10–12]. The results of the present study revealed that nearly all FET protocols were pretreated with GnRHa, indicating a higher live birth rate in 2020 group. Moreover, several trials assessed several types of GnRHa, while no significant correlation was found between pretreatment with and without GnRH agonist in terms of pregnancy rate after FET [12–14].

Regarding the limitation of the present study, this was a retrospective study and due to the COVID-19 pandemic, fewer patients could undergo FET. Therefore, the limited sample size limits the study findings. In this context, the current data were recorded in one single center; thus, the generalizability of the conclusions is limited.

## 5. Conclusions

Similar rates of clinical pregnancy, live birth, and implantation in the periods of 2019 and 2020 were found in the present study, which were consistent with previously reported findings [15]. An artificial protocol used for endometrial preparation, which is more stable and easier to guide patients to use it in the special periods, appeared to be a more appropriate choice for the COVID-19 lockdown period.

## Figures and Tables

**Table 1:** Baseline characteristics of patients in the 2019 and 2020 groups.

	2019 group	2020 group	*T*/*F* value	*P* value
Number of cases	34	59		
Age at FET (years old)	30.88 ± 4.04	32.83 ± 5.05	1.912	0.059
BMI (kg/m^2^)	22.75 ± 3.74	23.50 ± 3.43	0.968	0.336
Duration of infertility (years old)	4.02 ± 2.56	3.75 ± 3.23	0.423	0.673
Basal serum FSH (IU/L)	6.54 ± 2.87	7.40 ± 3.22	1.289	0.201
Basal serum LH (IU/L)	6.30 ± 4.60	5.67 ± 4.41	0.651	0.517
Basal serum E2 (pg/ml)	79.65 ± 101.23	64.46 ± 58.70	0.913	0.364
Serum total T (nmol/L)	0.46 ± 0.22	0.66 ± 1.02	1.096	0.276
Basal serum progesterone (ng/ml)	1.61 ± 3.03	1.80 ± 3.35	0.273	0.786
Basal AMH	4.80 ± 4.55	4.02 ± 3.41	0.929	0.355
Endometrial thickness on progesterone supplementation day (mm)	8.5 ± 1.3	8.2 ± 1.4	0.786	0.356
Artificial cycle/artificial cycles with down-regulated ratio	17.2% (5/29)	18% (9/50)		

Data are presented as mean ± standard deviation (SD); *P* < 0.05 indicates statistically significant differences.

**Table 2:** Comparison of the outcomes between 2019 and 2020 groups.

	2019 group	2020 group	*T*/*F* value	*P* value
Number of transferred embryos	1.47 ± 0.51	1.57 ± 0.50	0.907	0.367
Biochemical pregnancy rate (%)	70.6 (24/34)	72.9(43/59)	0.035	0.647
Implantation (%)	52(26/50)	48.4(45/93)	0.170	0.728
Clinical pregnancy rate (%)	61.8 (21/34)	61.0 (36/59)	0.005	0.892
Miscarriage rate (%)	23.8 (5/21)	19.4 (7/36)	0.149	0.699
Live birth rate (%)	47.1 (16/34)	49.2 (29/59)	0.038	0.681
Cancelled cycle rate (%)	0	28.39(23/82)	11.895	0.043

## Data Availability

The data used to support the findings of this study are available from the corresponding author upon reasonable request.
